# Supplementary material for: Hypoxia inducible factor signaling in breast tumors controls spontaneous tumor dissemination in a site-specific manner
Source: Commun Biol. 2021 Sep 23;4:1122. doi: 10.1038/s42003-021-02648-3 (PMC8460839; doi:10.1038/s42003-021-02648-3)
Supplement: Supplementary file 2 — Supplementary Information [file 42003_2021_2648_MOESM2_ESM.pdf]

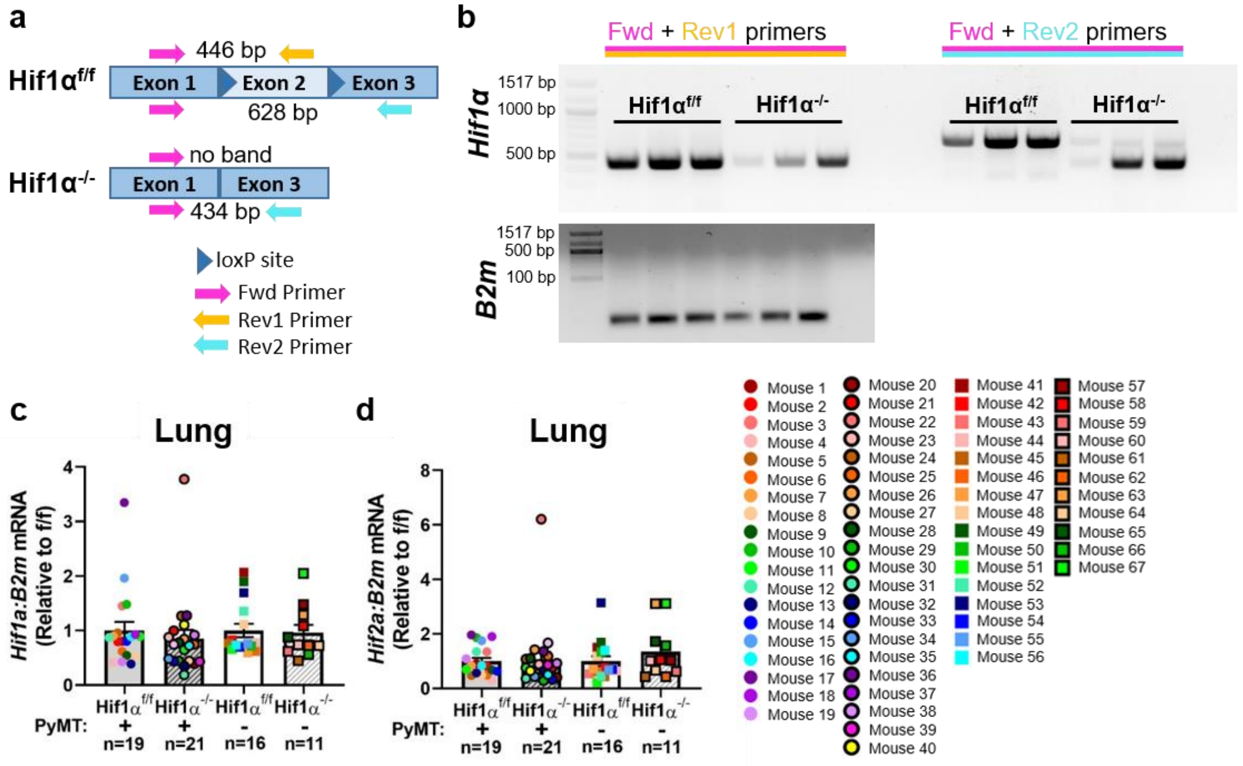

Supplementary Figure 1. Validation of mammary fat pad-specific *Hif1α* recombination. (a) Schematic of PCR-based validation of *Hif1α* locus recombination using two separate PCR reactions. cDNA was used as the input material for these reactions. Thus, no introns are included in the diagram. The loxP sites shown are for reference to indicate which exon is excised upon recombination. No loxP sequence is present in the mRNA/cDNA. **(b)** DNA electrophoresis gels of the PCR products from the reactions depicted in A. The lower intensity of the bands in the Hif1α$^{-/-}$ tumors from the "Fwd + Rev1" reaction, and the lower molecular weight of the band resulting from the "Fwd + Rev2" reaction indicate successful recombination. The residual band in the Hif1α$^{-/-}$ tumors from the "Fwd + Rev1" reaction is likely from non-recombined stromal cells present in the tumor. *B2m* was also amplified to control against differences in cDNA concentration. **(c, d)** Quantitative PCR analysis of *Hif1α* or *Hif2α* transcript, respectively,

compared to *B2m* from right lung homogenate RNA. Two-tailed Mann-Whitney test. Graphs represent mean per group and error bars represent s.e.m.

Supplementary Figure 2

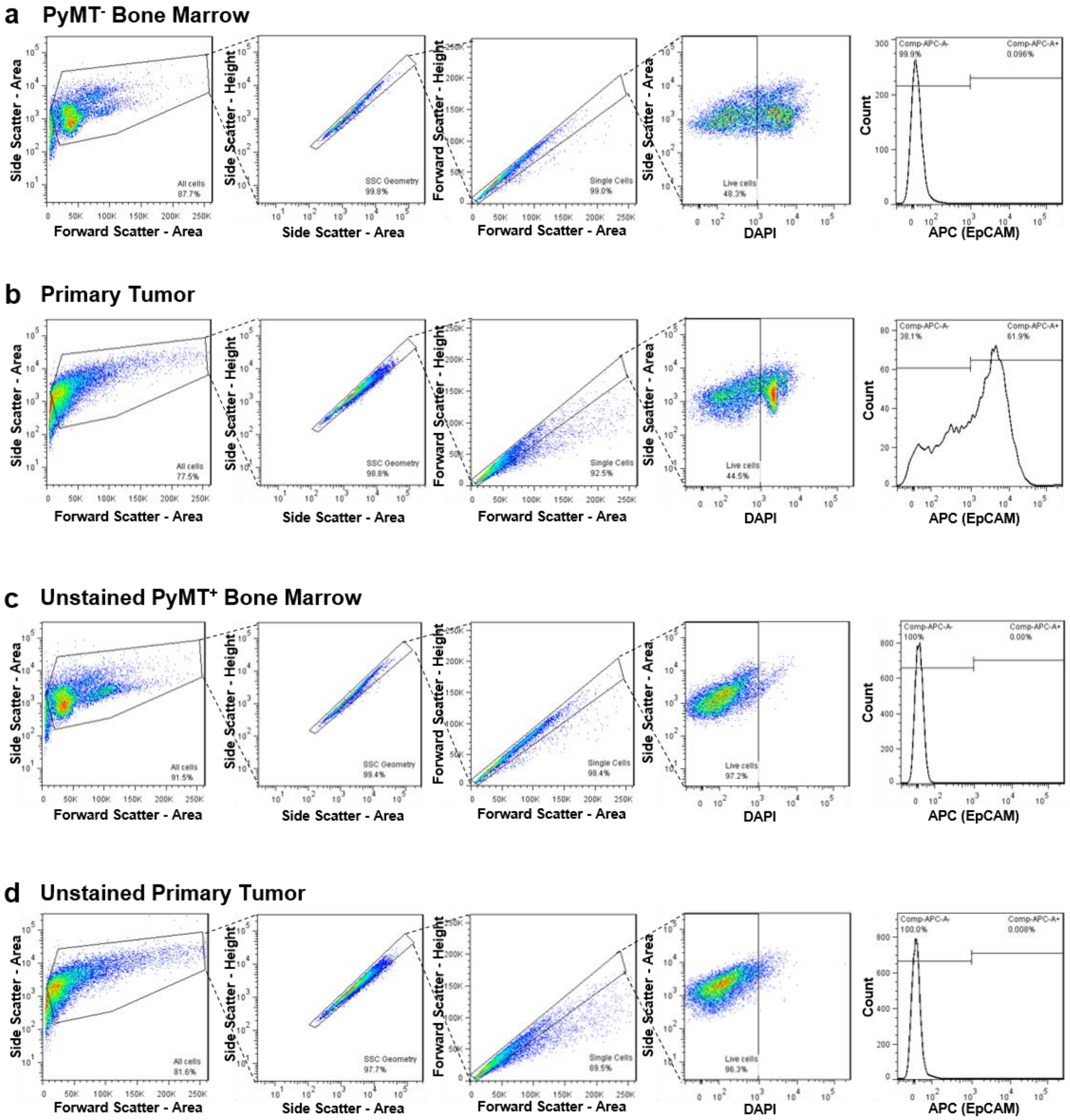

Supplementary Figure 2. Flow cytometry gating strategy. (a) Gating of PyMT^{-} (non-tumor bearing) bone marrow that acts as a negative control. **(b)** Gating of mechanically digested primary tumor cells that act as a positive control. **(c)** Gating of unstained PyMT^{+} bone marrow. **(d)** Gating of unstained primary tumor cells. In each case, cells were first gated based on forward and side scatter to gate out very small events that are likely debris. These events are next gated

on side and forward scatter geometry to identify single cells. These single cell events are next gated on DAPI intensity as a live-dead stain. $DAPI^-$ cells are then gated based on APC (EpCAM) intensity to detect tumor cells.

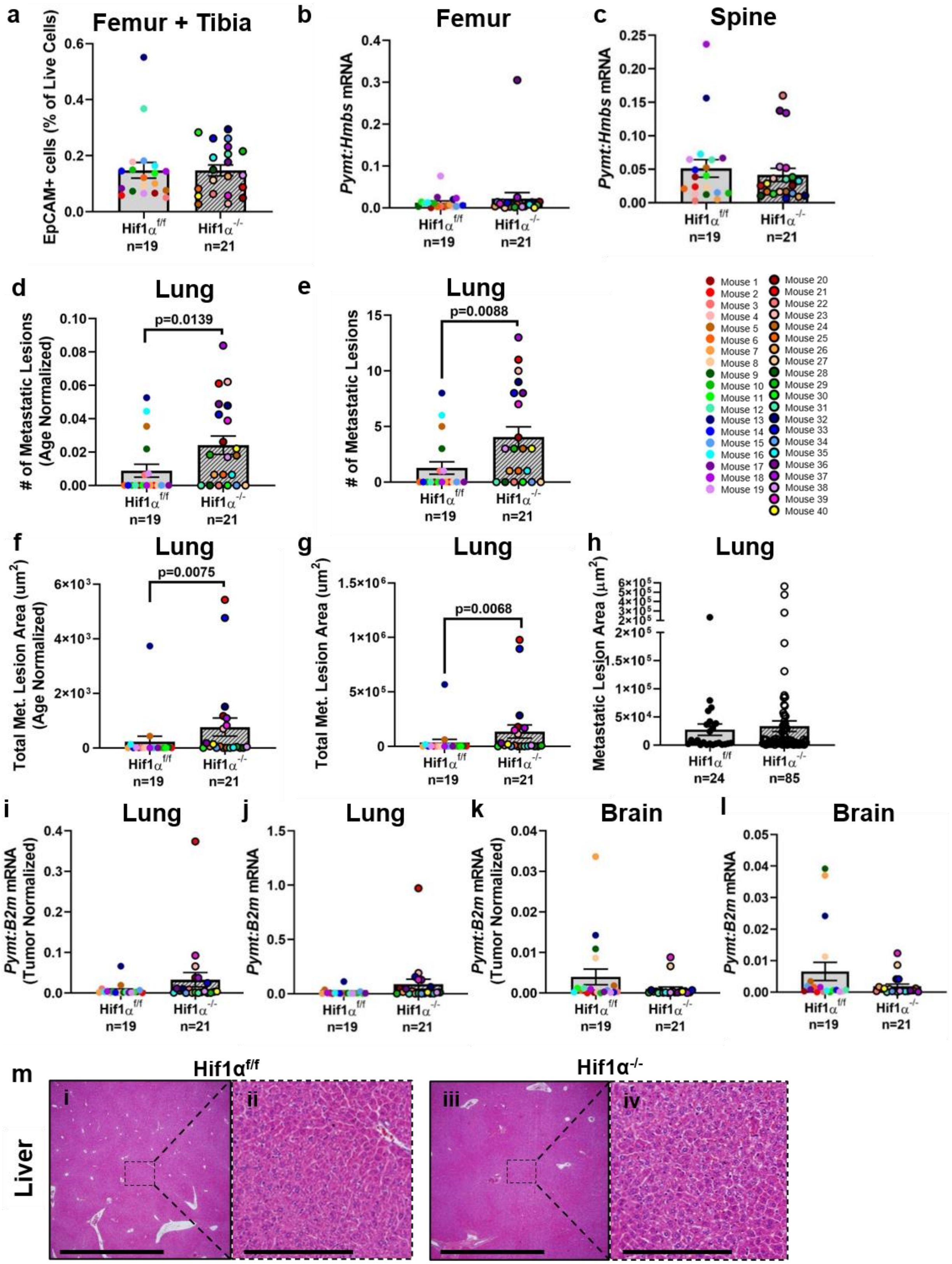
a
Femur + Tibia
EpCAM+ cells (% of Live Cells)
Hif1α f/f n=19
Hif1α -/- n=21
b
Femur
Pymt:Hmbs mRNA
c
Spine
d
Lung
# of Metastatic Lesions (Age Normalized)
p=0.0139
e
Lung
# of Metastatic Lesions
p=0.0088
Mouse 1 … Mouse 40
f
Lung
Total Met. Lesion Area (um2) (Age Normalized)
p=0.0075
g
Lung
Total Met. Lesion Area (um2)
p=0.0068
h
Lung
Metastatic Lesion Area (μm2)
Hif1α f/f n=24
Hif1α -/- n=85
i
Lung
Pymt:B2m mRNA (Tumor Normalized)
j
Lung
Pymt:B2m mRNA
k
Brain
Pymt:B2m mRNA (Tumor Normalized)
l
Brain
Pymt:B2m mRNA
m
Hif1αf/f
Hif1α-/-
Liver
i
ii
iii
iv

Supplementary Figure 3. Alternate normalization of tumor burden in bone and soft tissue sites from *Hif1α* knockout mice. (a) The percentage of $EpCAM^+$ cells, out of the total number of live cells, detected by flow cytometry analysis of left hindlimb bone marrow. Two-tailed Mann-Whitney test. **(b, c)** Quantitative PCR analysis of *Pymt* transcript compared to *Hmbs* from right femur or spinal midsection, respectively. Two-tailed Mann-Whitney test. **(d-g)** Total metastatic lesion number or area detected by histological analysis of H&E stained sections from the left lung. Numbers are normalized to the mouse's age at sacrifice (d, f) or left un-normalized (e, g). Two-tailed Mann-Whitney test. **(h)** Comparison of individual lesion areas detected from histological inspection of left lung sections. Two-tailed Mann-Whitney test. **(i-l)** Quantitative PCR analysis of *Pymt* transcript compared to *B2m* from right lung or brain. Numbers are normalized to total tumor weight (i, k) or left un-normalized (j, l). Two-tailed Mann-Whitney test. **(m)** Representative H&E stained images of the liver taken with 4x and 40x objectives. Scale bars represent 500 μm in 10x fields, and 200 μm in 40x fields. The 40x field is denoted with a dashed box in the 4x view. Graphs represents mean per group and error bars represent s.e.m.

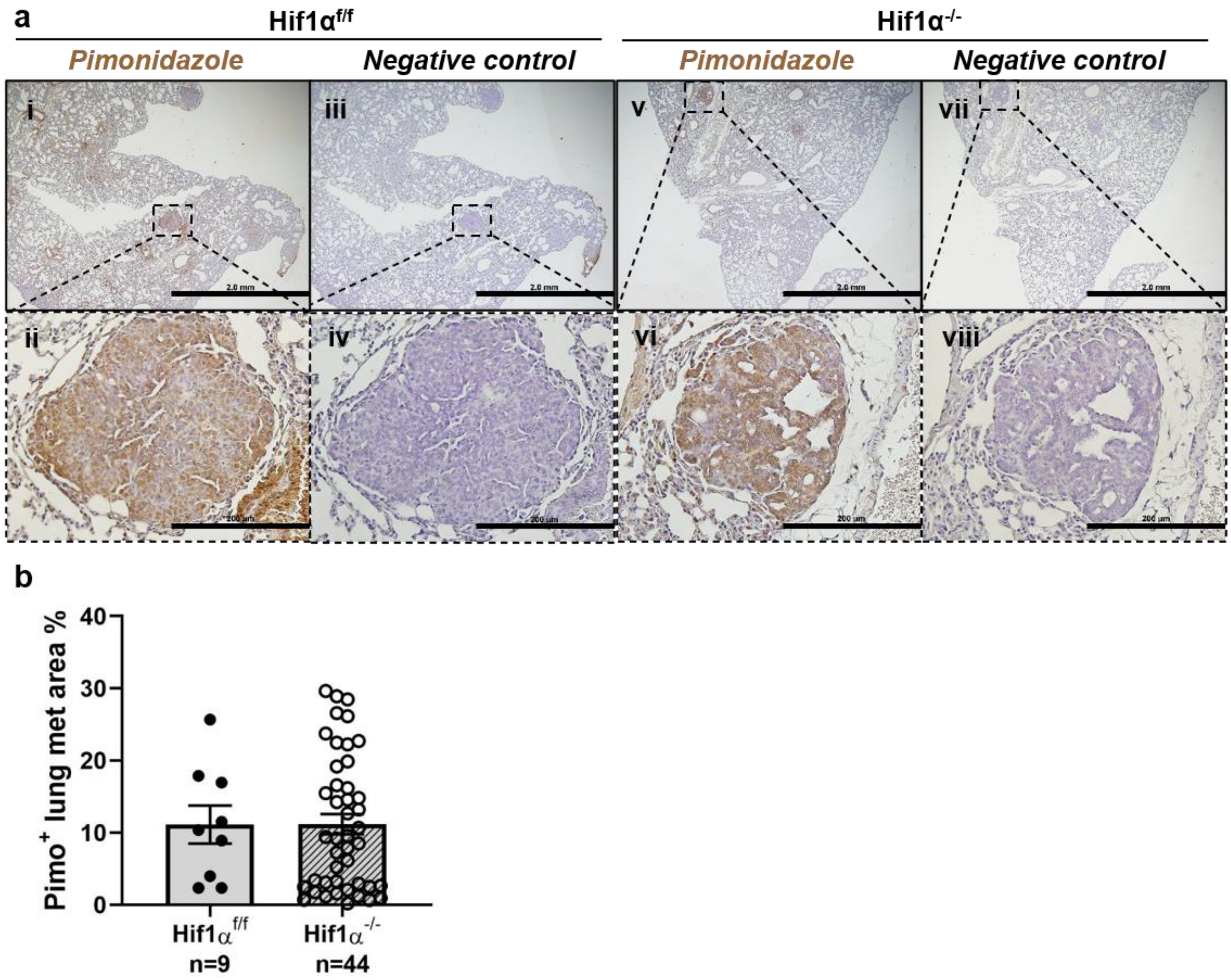

Supplementary Figure 4. ***Hif1α*** **deletion does not alter hypoxia of lung metastatic foci. (a)** Representative images of pimonidazole staining, taken with 4x and 40x objectives. The 40x field is denoted with a dashed box in the 10x view. Scale bars represent 2 mm in 4x fields, and 200 μm in 40x fields. Negative controls lacked the primary antibody incubation step. **(b)** Quantification of pimonidazole-positive area as a percentage of the lesion area. Two-tailed Mann-Whitney test. Graphs represent mean per group and error bars represent s.e.m.

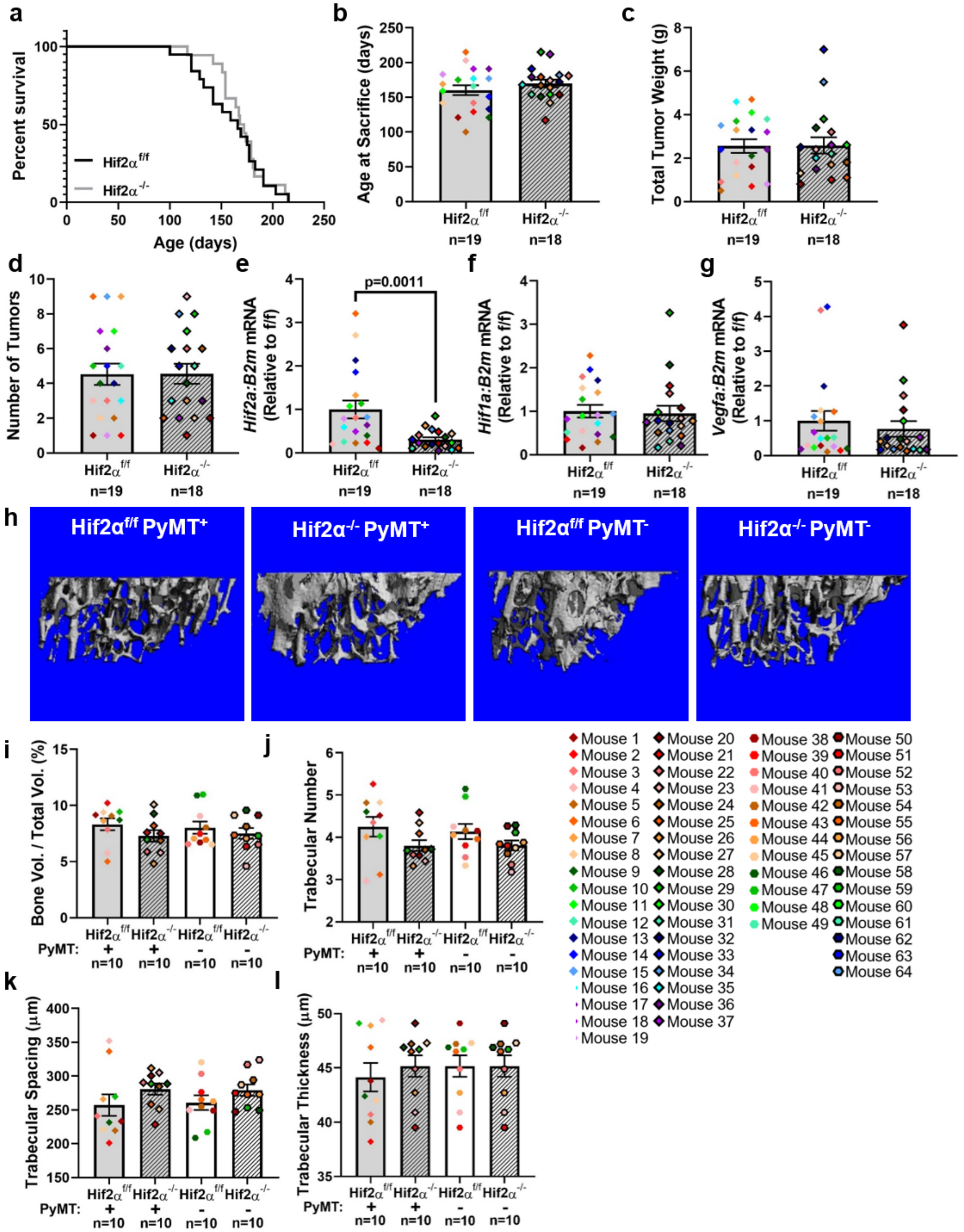
a
Percent survival
Age (days)
Hif2α f/f
Hif2α -/-
b
Age at Sacrifice (days)
c
Total Tumor Weight (g)
d
Number of Tumors
e
p=0.0011
Hif2a:B2m mRNA (Relative to f/f)
f
Hif1a:B2m mRNA (Relative to f/f)
g
Vegfa:B2m mRNA (Relative to f/f)
n=19
n=18
h
Hif2α f/f PyMT+
Hif2α -/- PyMT+
Hif2α f/f PyMT-
Hif2α -/- PyMT-
i
Bone Vol. / Total Vol. (%)
j
Trabecular Number
k
Trabecular Spacing (μm)
l
Trabecular Thickness (μm)
PyMT:
n=10

Supplementary Figure 5. Deletion of *Hif2α* does not alter total tumor burden, tumor growth kinetics, or trabecular bone parameters. (a) Survival analysis of Hif2α$^{f/f}$ PyMT^{+} and Hif2α$^{-/-}$ PyMT^{+} mice where endpoint represents sacrifice due to tumor size reaching collection threshold. Log-rank test. **(b-d)** Comparison of the age at sacrifice, total burden at sacrifice, and number of tumors collected per mouse. Two-tailed Mann-Whitney test. (**e-g)** Quantitative PCR analysis of *Hif2α*, *Hif1α*, and *Vegfa* expression compared to *B2m* from whole tumor homogenate RNA. Expression is normalized to the mean of the f/f control group. Two-tailed Mann-Whitney test. **(h)** Representative 3D renderings of microCT scans of the proximal metaphysis of the right tibia. (**i-l)** Quantification of bone volume as a percentage of total volume, trabecular number, trabecular spacing, and trabecular thickness. Two-tailed Mann-Whitney test against corresponding f/f control. Graphs represent mean per group and error bars represent s.e.m.

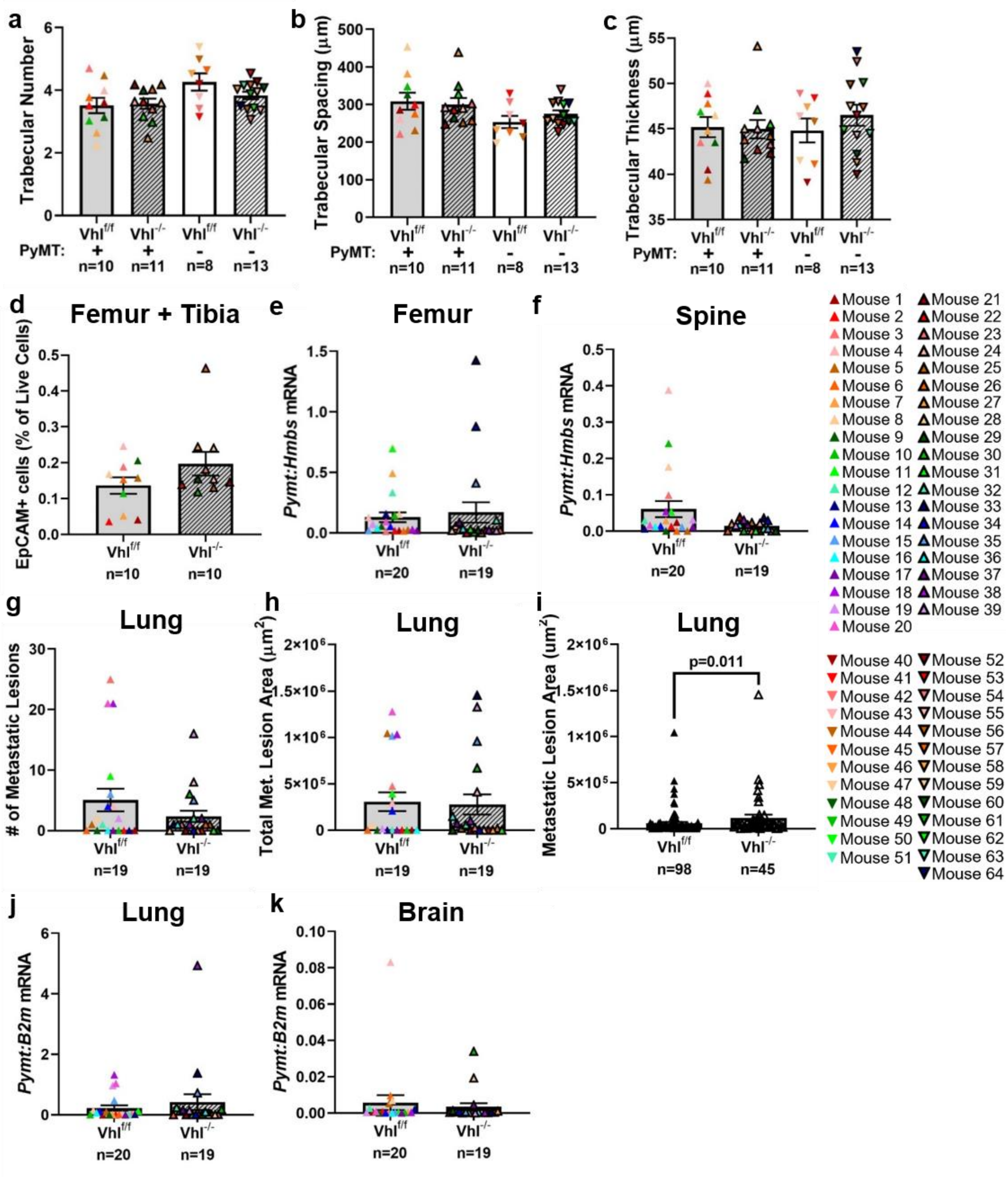

Supplementary Figure 6. Un-normalized tumor burden in bone and soft tissue sites from *Vhl* knockout mice. (a-c) Quantification of trabecular number, trabecular spacing, and trabecular thickness from microCT analysis of proximal tibia. Two-tailed Mann-Whitney test

against corresponding f/f control. **(d)** The percentage of $EpCAM^+$ cells, out of the total number of live cells, detected by flow cytometry analysis of left hindlimb bone marrow. Two-tailed Mann-Whitney test. **(e, f)** Quantitative PCR analysis of *Pymt* transcript compared to *Hmbs* from right femur or spinal midsection, respectively. Two-tailed Mann-Whitney test. **(g, h)** Total metastatic lesion number or area detected by histological analysis of H&E stained sections from the left lung. Two-tailed Mann-Whitney test. **(i)** Comparison of individual lesion areas detected from histological inspection of left lung sections. Two-tailed Mann-Whitney test. **(j, k)** Quantitative PCR analysis of *Pymt* transcript compared to *B2m* from right lung or brain, respectively. Two-tailed Mann-Whitney test. Graphs represent mean per group and error bars represent s.e.m.

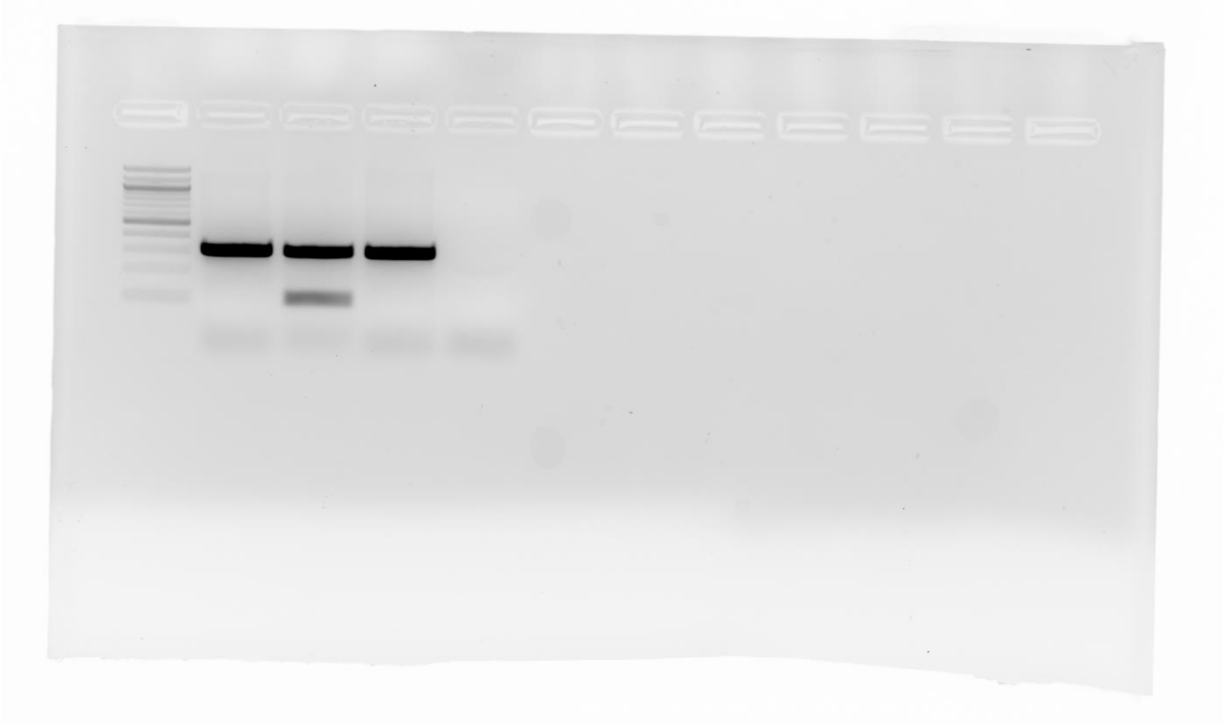

Supplementary Figure 7. Unedited Cre PCR gel image from Figure 1a.

Supplementary Figure 8

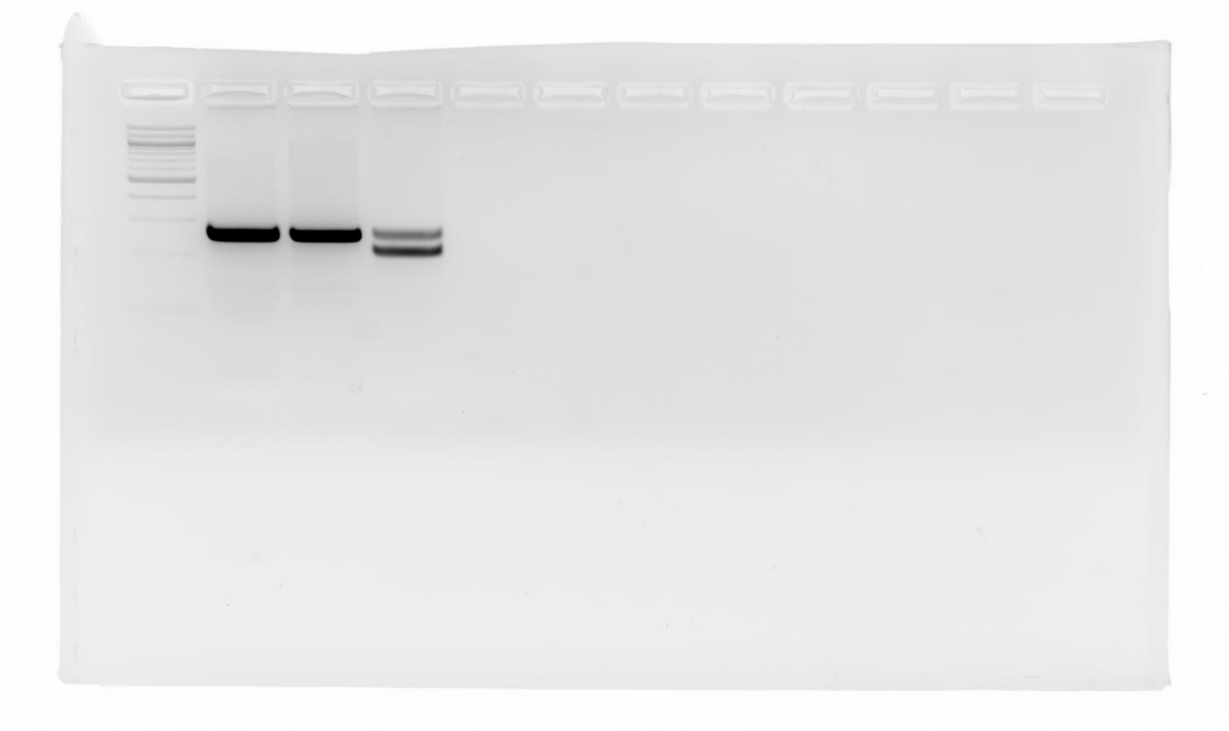

Supplementary Figure 8. Unedited Hif1α PCR gel image from Figure 1a.

Supplementary Figure 9

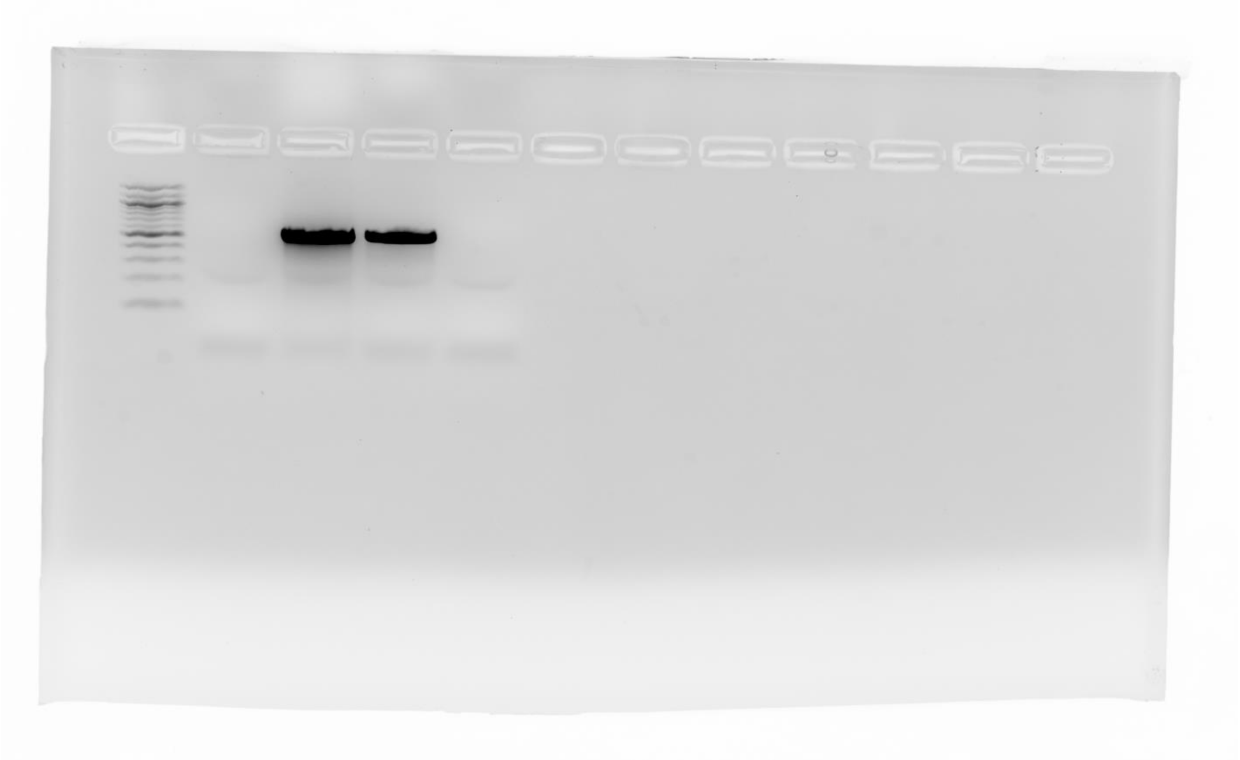

Supplementary Figure 9. Unedited PyMT PCR gel image from Figure 1a.

Supplementary Figure 10

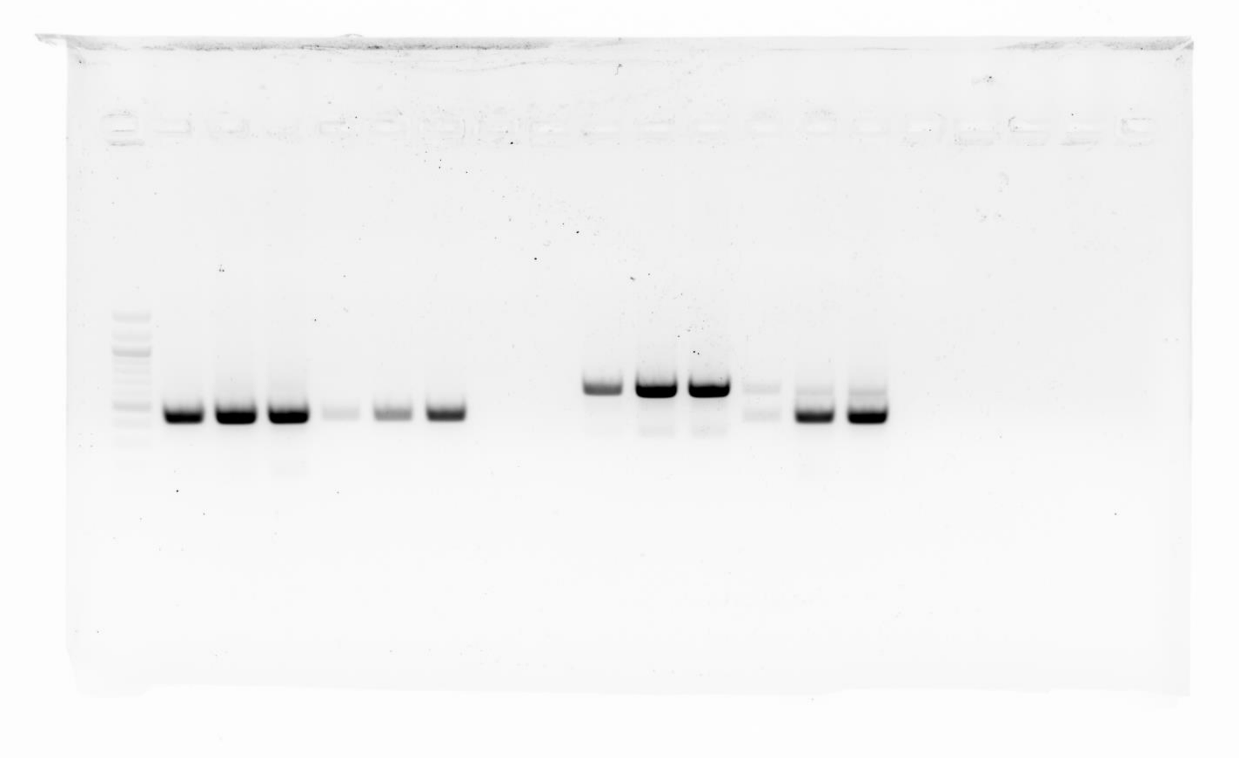

Supplementary Figure 10. Unedited tumor Hif1α recombination PCR gel image from Figure 2b.

Supplementary Figure 11

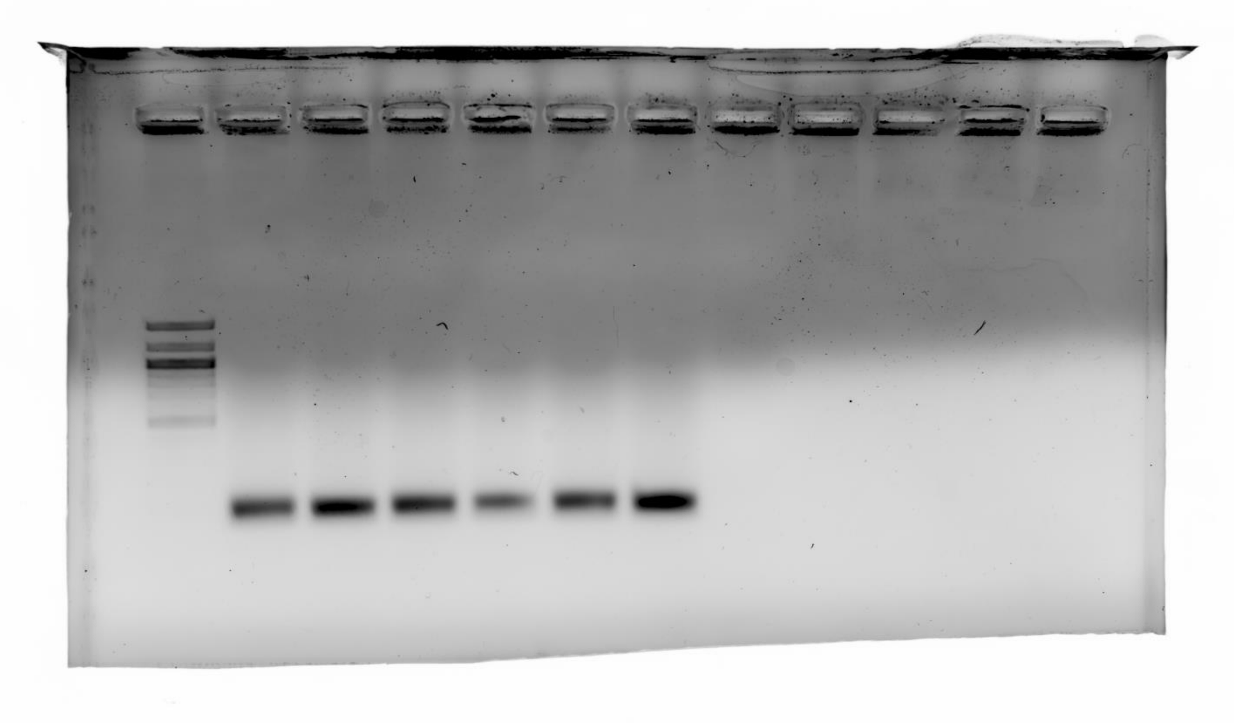

Supplementary Figure 11. Unedited tumor B2m PCR gel image from Figure 2b.

Supplementary Figure 12

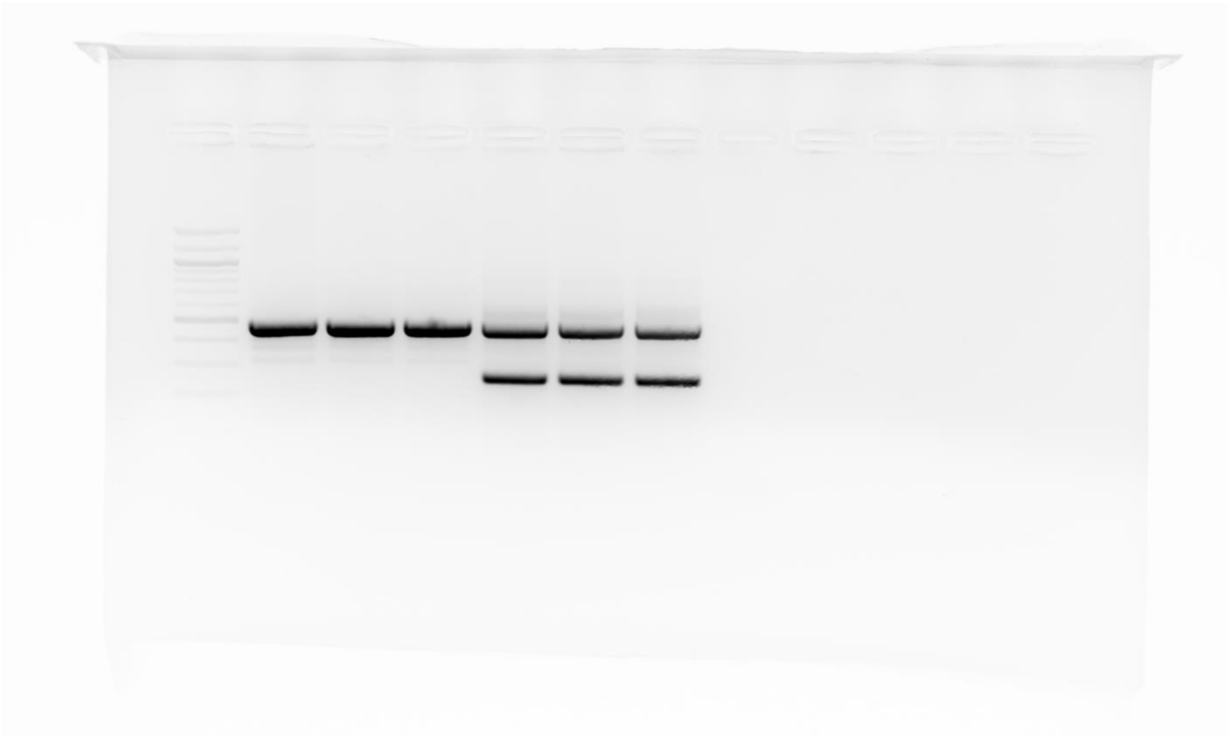

Supplementary Figure 12. Unedited tumor Vhl recombination PCR gel image from Figure 6f.
