# Supplementary material for: Hypoxia inducible factor signaling in breast tumors controls spontaneous tumor dissemination in a site-specific manner
Source: Commun Biol. 2021 Sep 23;4:1122. doi: 10.1038/s42003-021-02648-3 (PMC8460839; doi:10.1038/s42003-021-02648-3)
Supplement: Supplementary file 3 — Description of Additional Supplementary Files [file 42003_2021_2648_MOESM3_ESM.pdf]

Description of Additional Supplementary Files

File name: Supplementary Data 1

Description: All raw data underlying graphs.
