# Supplementary material for: Hypoxia inducible factor signaling in breast tumors controls spontaneous tumor dissemination in a site-specific manner
Source: Commun Biol. 2021 Sep 23;4:1122. doi: 10.1038/s42003-021-02648-3 (PMC8460839; doi:10.1038/s42003-021-02648-3)
Supplement: Supplementary file 5 — Reporting Summary [file 42003_2021_2648_MOESM5_ESM.pdf]

nature research

Corresponding author(s): Rachelle W. Johnson

Last updated by author(s): Aug 23, 2021

Reporting Summary

Nature Research wishes to improve the reproducibility of the work that we publish. This form provides structure for consistency and transparency in reporting. For further information on Nature Research policies, see our Editorial Policies and the Editorial Policy Checklist.

Statistics

For all statistical analyses, confirm that the following items are present in the figure legend, table legend, main text, or Methods section.

n/a	Confirmed	
[ ]	[x]	The exact sample size (*n*) for each experimental group/condition, given as a discrete number and unit of measurement
[ ]	[x]	A statement on whether measurements were taken from distinct samples or whether the same sample was measured repeatedly
[ ]	[x]	The statistical test(s) used AND whether they are one- or two-sided<br>*Only common tests should be described solely by name; describe more complex techniques in the Methods section.*
[x]	[ ]	A description of all covariates tested
[ ]	[x]	A description of any assumptions or corrections, such as tests of normality and adjustment for multiple comparisons
[ ]	[x]	A full description of the statistical parameters including central tendency (e.g. means) or other basic estimates (e.g. regression coefficient) AND variation (e.g. standard deviation) or associated estimates of uncertainty (e.g. confidence intervals)
[ ]	[x]	For null hypothesis testing, the test statistic (e.g. *F*, *t*, *r*) with confidence intervals, effect sizes, degrees of freedom and *P* value noted<br>*Give P values as exact values whenever suitable.*
[x]	[ ]	For Bayesian analysis, information on the choice of priors and Markov chain Monte Carlo settings
[x]	[ ]	For hierarchical and complex designs, identification of the appropriate level for tests and full reporting of outcomes
[ ]	[x]	Estimates of effect sizes (e.g. Cohen's *d*, Pearson's *r*), indicating how they were calculated

Our web collection on statistics for biologists contains articles on many of the points above.

Software and code

Policy information about availability of computer code

Data collection	No code or software was used in data collection for this study.
Data analysis	SPOT software (Diagnostic Instruments Inc) was used for quantitative histological analysis of tumor area in lungs. ImageJ software was used for quantification of immunohistochemistry staining. Graphpad Prism software (version 8) was used for statistical analyses.

For manuscripts utilizing custom algorithms or software that are central to the research but not yet described in published literature, software must be made available to editors and reviewers. We strongly encourage code deposition in a community repository (e.g. GitHub). See the Nature Research guidelines for submitting code & software for further information.

Data

Policy information about availability of data

All manuscripts must include a data availability statement. This statement should provide the following information, where applicable:

- Accession codes, unique identifiers, or web links for publicly available datasets
- A list of figures that have associated raw data
- A description of any restrictions on data availability

Raw data underlying figures can be found in the Supplementary Dataset. Unedited DNA gel images are available in the Supplementary Information (Supp. Fig. 7-12). Any other data that support the findings of this study are available from the corresponding author, R.W.J., upon reasonable request.

Field-specific reporting

Please select the one below that is the best fit for your research. If you are not sure, read the appropriate sections before making your selection.

- [x] Life sciences
- [ ] Behavioural & social sciences
- [ ] Ecological, evolutionary & environmental sciences

For a reference copy of the document with all sections, see nature.com/documents/nr-reporting-summary-flat.pdf

Life sciences study design

All studies must disclose on these points even when the disclosure is negative.

Sample size	We previously consulted with a biostatistician within the Department of Radiation Oncology at Stanford University who performed power calculations for us. The power calculations were based on previously published in vivo studies (DOI 10.1016/j.ccr.2007.12.003). For the transgenic mouse lines, a sample size of n=10 mice/group will provide 85% power in a two-sided t test with an alpha level 0.05 with a variation of 8.5 and an expected difference of 12. Thus, we sought to collect a minimum of n=10 mice/group to ensure adequate statistical analysis at end point for these experiments.
Data exclusions	No data was excluded from analysis.
Replication	All qPCR analysis was performed with 3 technical replicates, and all analyses involving mouse samples were performed on all available samples. The exception is Figure 4a, where qPCR was performed with technical duplicates and only n=3 mouse tumors were analyzed for each group. All attempts at replication were successful.
Randomization	Since this study involves a spontaneous genetic mammary carcinoma model, mice were not randomly assigned to groups. Both tumor bearing (PyMT+) and non-tumor bearing (PyMT-) mice were collected.
Blinding	Histological analysis and quantification of lung tumor burden were performed by certified veterinary pathologists that were blinded to the mouse genotype. IHC and immunofluorescent imaging and quantification was performed by authors blinded to the genotype.

Reporting for specific materials, systems and methods

We require information from authors about some types of materials, experimental systems and methods used in many studies. Here, indicate whether each material, system or method listed is relevant to your study. If you are not sure if a list item applies to your research, read the appropriate section before selecting a response.

Materials & experimental systems

n/a	Involved in the study	
☐	☒	Antibodies
☒	☐	Eukaryotic cell lines
☒	☐	Palaeontology and archaeology
☐	☒	Animals and other organisms
☒	☐	Human research participants
☒	☐	Clinical data
☒	☐	Dual use research of concern

Methods

n/a	Involved in the study	
☒	☐	ChIP-seq
☐	☒	Flow cytometry
☒	☐	MRI-based neuroimaging

Antibodies

Antibodies used	Hypoxyprobe rabbit antipimonidazole antibody (PAb2627AP, Hypoxyprobe-1 Omni Kit, Hypoxyprobe, Inc, catalog number HP3-1000Kit), biotinylated goat-anti-rabbit IgG, (Vector, catalog number BA-1000), anti-EpCAM APC antibody (BD Pharmingen, catalog number 563478), Ki-67 (Catalog #12202S, Cell Signaling Technology, Danvers, MA), CD4 (Invitrogen, clone 4SM95), CD8 (Invitrogen, clone 4SM16), ImmPRESS HRP Goat anti-rat IgG (Vector Laboratories)
Validation	The Hypoxyprobe system is well validated and widely used. Studies utilizing this system have been published previously in Nature group journals (Johnson RW et al. Induction of LIFR confers a dormancy phenotype in breast cancer cells disseminated to the bone marrow. Nat Cell Biol. 2016 Oct;18(10):1078-1089. doi: 10.1038/ncb3408. Epub 2016 Sep 19.).<br>The EpCAM-APC antibody used is tested for reactivity with mouse EpCAM in flow cytometry applications by the manufacturer, and we validated that it bound to tumor cells and not bone marrow cells, as shown in the supplemental data.<br>The Ki-67 antibody used has been validated by both the manufacturer and the Vanderbilt University Medical Center Translational Pathology Shared Resource.<br>The CD4 and CD8 antibodies have been tested by immunohistochemistry of formalin-fixed paraffin embedded mouse tissue by the manufacturer, and use of these antibodies with the Opal 7-Color Manual IHC Kit was validated prior to use in this study.

Animals and other organisms

Policy information about studies involving animals; ARRIVE guidelines recommended for reporting animal research

Laboratory animals	Hif1a-/-, Hif2a-/-, and Vhl-/- mice were generated by breeding a transgenic mice with loxP sites flanking both alleles of either Hif1α exon 2 (Jackson Laboratory Stock No. 007561, C57/B6 background), Hif2α exon 2 (Jackson Laboratory Stock No. 008407, C57/B6 background), or Vhl exon 1 (Jackson Laboratory Stock No. 012933, C57/B6 background), with transgenic mice expressing Cre recombinase downstream of mammary tumor virus long terminal repeats (MMTV-LTR) (Jackson Laboratory Stock No. 003553, C57/B6 background). The progeny from this cross were then bred with transgenic mice expressing the polyoma middle T (PyMT) oncoprotein under the MMTV-LTR (Jackson Laboratory Stock No. 022974, C57/B6-FVB mixed background). These mice developed tumor around 8 weeks of age and were collected at around 22-32 weeks of age when tumor size met collection criteria. All mice used in the study were female.
Wild animals	This study did not involve wild animals.
Field-collected samples	This study did not involve samples collected from the field.
Ethics oversight	All experiments were performed following the relevant guidelines and regulations of the Animal Welfare Act and the Guide for the Care and Use of Laboratory Animals and were approved by the Institutional Animal Care and Use Committee (IACUC) at Vanderbilt University.

Note that full information on the approval of the study protocol must also be provided in the manuscript.

Flow Cytometry

Plots

Confirm that:

- [x] The axis labels state the marker and fluorochrome used (e.g. CD4-FITC).
- [x] The axis scales are clearly visible. Include numbers along axes only for bottom left plot of group (a 'group' is an analysis of identical markers).
- [x] All plots are contour plots with outliers or pseudocolor plots.
- [x] A numerical value for number of cells or percentage (with statistics) is provided.

Methodology

Sample preparation	The epiphyses of the femur and tibia from one hindlimb were cut and the bones were flushed using centrifugation to obtain the bone marrow. The bone marrow was filtered through a 40μm cell strainer to separate the cells from bone debris. A primary tumor sample was mechanically digested and filtered through a 40μm cell strainer to separate the cells from debris, and used as a positive control to establish the APC-EpCAM gate. Cells were suspended in red blood cell lysis buffer for 5 minutes on ice, spun down, and washed twice with PBS. 2 million cells were stained in 100ul of 1% BSA in PBS with 100ng EpCAM antibody (BD Pharmingen, catalog number 563478) for 1 hour at 4 °C in the dark. Cells were washed with PBS and resuspended in PBS and 0.5ng DAPI for 15 min on ice. Cells were then washed once more with PBS before analysis.
Instrument	Hif1a f/f and Hif1a-/- samples were analyzed on a 3-laser LSRII (BD) instrument. Hif2a f/f and Hif2a-/- samples, as well as Vhl f/f and Vhl -/- samples were analyzed on the 5-laser LSRII (BD) instrument.
Software	FlowJo software was used for flow cytometry data analysis.
Cell population abundance	EpCAM+ cells were rare in the bone marrow samples, making up less than 1% of the live (DAPI-) cells. While some bone marrow cells (from non-tumor bearing PyMT- mice) were captured in the EpCAM+ gate, tumor cell enrichment was detected by comparison of the f/f and -/- groups, controlling for any inclusion of background bone marrow cells, which would be equally abundant across samples.
Gating strategy	Cells were first gated based on forward and side scatter to gate out very small events that are likely debris. These events are next gated on side and forward scatter geometry (side scatter area vs. side scatter height, then forward scatter area vs. forward scatter height) to identify single cells. Cells that deviated significantly from the straight 45 degree line on these plots were excluded from further analysis. These single cell events are next gated on DAPI intensity as a live-dead stain. DAPI- cells are then gated based on APC (EpCAM) intensity to detect tumor cells. APC gate was set to capture the majority of EpCAM+ cells in a positive-control tumor cell sample, while capturing less than 0.1% of the negative control non-tumor bearing PyMT- bone marrow sample.

- [x] Tick this box to confirm that a figure exemplifying the gating strategy is provided in the Supplementary Information.
